# A General Model of Dissonance Reduction: Unifying Past Accounts via an Emotion Regulation Perspective

**DOI:** 10.3389/fpsyg.2020.540081

**Published:** 2020-11-11

**Authors:** Sebastian Cancino-Montecinos, Fredrik Björklund, Torun Lindholm

**Affiliations:** ^1^Division of Personality, Social, and Developmental Psychology, Department of Psychology, Stockholm University, Stockholm, Sweden; ^2^Division of Personality and Social Psychology, Department of Psychology, Lund University, Lund, Sweden

**Keywords:** dissonance theory, dissonance reduction, emotion regulation, appraisal theory, coping

## Abstract

Cognitive dissonance has been studied for more than 60 years and many insightful findings have come from this research. However, some important theoretical and methodological issues are yet to be resolved, particularly regarding dissonance reduction. In this paper, we place dissonance theory in the larger framework of appraisal theories of emotion, emotion regulation, and coping. The basic premise of dissonance theory is that people experience negative affect (to varying degrees) following the detection of cognitive conflict. The individual will be motivated to alleviate these emotional reactions and could do so by reducing dissonance in some manner. We argue that detection of dissonance will follow the same principles as when people interpret any other stimuli as emotionally significant. Thus, appraisal theory of emotion, which argues that emotions are generated via the cognitive evaluation of surrounding stimuli, should be applicable to the dissonance-detection process. In short, we argue that dissonance-reduction strategies (attitude change, trivialization, denial of responsibility, etc.) can be understood as emotion-regulation strategies. We further argue that this perspective contributes to reconciling fragmented (and sometimes contrary) viewpoints present in the literature on dissonance reduction. In addition to proposing the general model of dissonance reduction, we illustrate at the hand of empirical data how research on dissonance reduction can be performed without relying on experimental paradigms that focus on a specific reduction strategy.

## Introduction

In this paper, we present a novel approach for how to reconcile previous ideas and findings related to dissonance reduction in a more inclusive model. The first step for such reconciliation must make use of an *a priori* approach to the problem, where the theoretical groundwork for the new model is established. We first describe dissonance theory and review some of the major views on dissonance reduction. Later, we outline our theoretical account of dissonance reduction (based on Festinger’s original formulation from 1957) and show how past ideas of dissonance reduction can be understood under a broader model of emotion regulation. Thereafter, we offer some ideas for the potential correspondence between specific emotions and dissonance-reduction strategies. We will also illustrate the feasibility of the emotion regulation perspective by re-analyzing data from a typical dissonance study. Lastly, we offer an outlook for the continued theoretical development of the dissonance-reduction process. Overall, a key assumption in this paper is that dissonance reduction is a pluralistic process both in terms of emotional reactions and dissonance-reduction strategies.

### The Original Dissonance Theory and Related Research

In Festinger’s original formulation of dissonance theory the basic premise is that (a) people will experience psychological discomfort (i.e., negative affect) when related cognitions are perceived to be in conflict (i.e., dissonant); (b) this will motivate people to reduce the aversive feeling and restore consonance; and (c) people will avoid information and situations that could increase that specific dissonance (Festinger, 1957, p. 3). An example of cognitive conflict (or dissonance) is when individuals realize that their current behavior is contradicting a strongly held attitude–for instance, having pork chops at a family dinner while holding strong negative attitudes toward the meat industry. In most situations, however, there exists both dissonant and consonant cognitions. For example, whereas a nice family dinner might be consonant with one’s social goals, the meal served may be dissonant with one’s attitudes/values. Consequently, Festinger defined the magnitude of dissonance as the proportion of dissonant to consonant cognitions, and stated that important cognitions will have more weight in this calculation. What might eventually tip the scale toward more dissonance, in our example, is that for this individual the negative attitude toward the meat industry might be more important than having a nice family dinner. The cognition that is most resistant to change is called the *generative cognition* (Beauvois and Joule, 1996), and other cognitions are evaluated in terms of their relationship to this. Festinger assumed three major manners in which an individual could reduce dissonance: (1) change one of the dissonant cognitions (e.g., attitude change); (2) add consonant cognitions so that the overall inconsistency decreases (e.g., seeking information that explains one’s inconsistent behavior); and (3) decrease the importance of the cognitions in the dissonant situation (e.g., trivializing the dissonant behavior, or trivializing the importance of the attitude).

An array of different situations can be understood from the rather simple premises of dissonance theory, and in each of these situations there are many ways of reducing dissonance. The most common experimental paradigm in dissonance research is the *induced-compliance paradigm*, in which individuals are asked (under the perception of free choice) to write a counter-attitudinal essay. A well-known effect of inducing dissonance this way is that people tend to change their attitude as a way of reducing dissonance. That is, they become more positively tuned to something toward which they previously held a negative attitude (Festinger and Carlsmith, 1959). Another common way to investigate dissonance theory is by having people make difficult choices between equally attractive/unattractive options. This is called the *free-choice paradigm* and the typical prediction is that individuals will like the chosen option (vs. the non-chosen option) more after making the decision. This is called the spreading-of-alternatives effect and is thought to shield against post-decisional regret (Brehm, 1956). In the *effort-justification paradigm*, researchers study situations where people voluntarily engage in unpleasant behavior in order to reach a higher goal. The usual effect is that people begin to value the goal more, the more unpleasant the behavior–to guard against post-behavioral regret (Aronson and Mills, 1959). A paradigm developed from the self-consistency framework of cognitive dissonance theory (Aronson, 1992), the *induced-hypocrisy paradigm*, studies people’s reactions after their realization of their own hypocrisy. Here, people are usually asked to publicly endorse a pro-social/pro-environmental issue. Then, the experimenter asks them to recall a time in which they themselves did not follow their own endorsements. After this manipulation, people tend to engage in compensatory behavior as a way to make amends for their hypocrisy (Stone et al., 1994). A fifth way of testing dissonance theory is to present belief dilemmas to individuals. In this experimental set-up, called the *belief-disconfirmation paradigm*, individuals are confronted with information counter to their beliefs. Researchers have found that people usually deal with these dilemmas by seeking support from those who share one’s beliefs, but also by refuting and/or misperceiving/misinterpreting the new information (see e.g., Gawronski et al., 2014). Lastly, in the *selective-exposure paradigm*, (based on the notion that people seek desirable outcomes, and avoid undesirable ones; Mills, 1999) people are asked to browse through newspapers about different topics. The prediction is that people will attend more to news that reinforces their pre-existing opinions, and they will try to avoid contradictory information, in order to avoid potential belief dilemmas (i.e., cognitive dissonance, see Table 1).

**TABLE 1:** List of experimental paradigms in dissonance research.

Experimental set-up	Experimental task	Most common outcome variable
Induced-compliance paradigm	Write a counter-attitudinal essay	Attitude change
Free-choice paradigm	Make decision between equal alternatives	Spreading of alternatives
Effort-justification paradigm	Engage in dissonant behavior to reach goal	Increased liking of the goal
Induced-hypocrisy paradigm	Public endorsement of pro-social issue	Behavioral change
Belief-disconfirmation paradigm	Confronted with information counter to belief	Refutation or misinterpretation of counter-belief information
Selective-exposure paradigm	Information search	Pattern of information search

Although there has been a very large emphasis on the attitude-change effect, and predominantly within the induced-compliance paradigm (see e.g., Devine et al., 1999; Harmon-Jones et al., 2009 for a similar concern), the role of other reduction strategies has also received some attention. For example, studies have documented strategies such as *denial of responsibility* (Gosling et al., 2006), *act rationalization* (Beauvois et al., 1993), *behavioral change* (Stone et al., 1994), *trivialization* (Simon et al., 1995), *attitude bolstering* (Sherman and Gorkin, 1980), and simply *forgetting* about the dissonant situation (Zanna and Aziza, 1976). Hence, there are several different notions on how the dissonance-reduction process might work, but no account has yet managed to encapsulate the widespread findings in the literature (see also McGrath, 2017; Vaidis and Bran, 2018, on this point).

## Dissonance Reduction Strategies and Motivations: Current Accounts

Festinger did not explicitly state when and where one strategy would be preferred over another, merely that the preferred reduction strategy depends on many variables (e.g., situation, personality, habitual behavior, specific dissonant cognition, etc.). Although Festinger was somewhat vague on the issue of dissonance reduction, his theoretical model laid out enough ideas for subsequent researchers to build on. For example, regarding belief dilemmas, Abelson (1959) posited that the more difficult the dilemma the more people would increase their level of effort to reduce inconsistencies (from denial to transcendence, i.e., seeing the big picture). Researchers focusing on induced compliance assume that dissonance reduction is a function of the importance of the dissonant cognitions (e.g., Hardyck and Kardush, 1968; Leippe and Eisenstadt, 1999). For unimportant cognitions, simply forgetting about it would be the predicted outcome. For moderately important cognitions, people might change their attitude, while for highly important cognitions the predicted outcome would be mental restructuring (e.g., reaffirming one’s original viewpoint via attitude bolstering). In their functionalist view, Kelman and Baron (1968) argue that inconsistencies related to the same goal (e.g., eating meat while being vegan) activate reduction mechanisms (e.g., attitude change or bolstering), whereas inconsistencies related to different goals (e.g., being a present father but also being a productive scholar) activate maintenance mechanisms (e.g., transcendence). Furthermore, when inconsistencies relate to short-term goals (e.g., eating sweets before dinner) people might simply try to forget the conflict, however, when inconsistencies relate to long-term goals (e.g., being a good husband and father) people tend to confront the conflict (via, e.g., transcendence). Concerning effort justification, Weick (1968) argues that the social context in which the dissonance occurs may determine the reduction strategy. For instance, dissonant behavior in the presence of friends and family (vs. alone) might bring about self-justification or vindication since the undoing of the dissonant behavior might be embarrassing. Within a developmental viewpoint, Kaplan and Crockett (1968) argue that cognitive complexity determines the reduction strategy. For example, due to lack of cognitive complexity, children’s reduction strategies are often rather primitive (e.g., denial), whereas adults are more refined (e.g., rationalization). More broadly, it has been suggested that dissonance reduction works as an exclusive switch (Simon et al., 1995), meaning that people will engage in only *one* dissonance-reduction strategy at a time, choosing whichever alternative is made available to them. For instance, when the cognitive conflict is made highly salient, or self-affirmation is readily available, trivialization will be more likely.

Several theorists have also pointed to motivations other than consonance restoration that may underlie dissonance reduction. Beauvois and Joule (1996, 1999) argue that the reduction process is about rationalizing a prior commitment to a behavior rather than restoring consonance. The self-affirmation viewpoint (Steele and Liu, 1983; Aronson et al., 2019) suggests that dissonance reduction functions as a way of restoring one’s self- image. A third notion questioning mere consonance motivation, the new look model (Cooper and Fazio, 1984), argues that dissonance reduction functions as a way of lessening aversive consequences when people feel personally responsible for having caused these consequences (see also Cooper, 2007, 2019). Finally, the self-consistency model (Aronson, 1969, 1992, 1999) holds, as does the original theory, that people seek consonance, however only when cognitive conflicts threaten self-integrity.

Despite the vast amount of research spawned by dissonance theory, there is to date no general model of dissonance reduction. A possible explanation for this could be that dissonance theory has gone through a series of reformulations (e.g., Aronson, 1969, 1992; Steele and Liu, 1983; Cooper and Fazio, 1984) emphasizing different aspects of the dissonance-reduction process. Another explanation might be that many previous ideas have been closely tied to their specific research paradigm (induced compliance, effort justification, induced hypocrisy, etc.), resulting in rather narrow models. This current state of affairs makes it difficult to reconcile the different approaches within a single theoretical account. We believe, however, that a change in perspective could make this challenging task slightly easier. Before proposing a broad model of dissonance-reduction, we will point to a critical theoretical issue in past research.

### Returning to the Emotional Component of Dissonance Theory

Past accounts of dissonance reduction have identified several different factors influencing dissonance reduction (e.g., the type of cognitions in conflict, situational circumstances, influence of other people, individual differences, personal goals, etc.). However, these accounts seem to downplay the main premise of Festinger’s formulation, namely that cognitive conflict will produce a negative emotional state that motivates individuals to attend to the situation and try to resolve the conflict (see also Devine et al., 1999; McGregor et al., 1999, 2019; Harmon-Jones et al., 2009, who argue for more focus on the emotional aspect of dissonance theory). Given that a negative emotional state is a basic premise of dissonance theory, accepted by all previous accounts of dissonance reduction and all major reformulations, it seems evident that an emotion-regulation account of dissonance reduction could be used to develop an encompassing model of the different research paradigms and findings (see also Cancino-Montecinos et al., 2018). To illustrate, assuming that dissonance emerges when self-integrity is threatened, and the subsequent reduction strategies assist in restoring the self-concept, the reduction process would be based on an emotional reaction to a perceived threat to the self. Similarly, if dissonance reduction is used as a rationalization of past behavior, or to lessen aversive consequences, it is the emotional reaction to the cognitive conflict that initiates these processes. Thus, regardless of the set of specific circumstances believed to be necessary to produce dissonance (specific situation, presence of others, goal conflict, etc.), the dissonance-reduction process always begins with an emotional reaction and unfolds to regulate the negative emotions resulting from the cognitive conflict. Whether people do this via restoration of consonance, rationalization of behavior, self-affirmation, or lessening of aversive consequences, will be determined by factors such as the situation in which the dissonance arose, the individual’s specific repertoire of reduction strategies, or habitual responses. Thus, with an emotion-regulation approach for understanding dissonance reduction, different notions and ideas on specific motives and strategies can be covered within a single model. Consequently, we suggest that framing dissonance reduction as emotion regulation is the first constructive step toward building a general model.

## A New Perspective on Dissonance Processes: Appraisal, Emotion Regulation, and Coping

Appraisal theories of emotion (e.g., Scherer, 2009; Moors et al., 2013) hold that the emotions following from a specific event are products of the cognitive evaluation of that situation. First, the individual makes a quick assessment of the stimuli’s relevance for ongoing goals. This assessment occurs at low-level processing, in which the stimuli’s novelty (familiar or unfamiliar situation) and intrinsic meaning (intrinsic pleasantness or unpleasantness) are classified. Subsequent processes occur at a higher cognitive level and involve evaluation of the stimuli in relation to implications for goals and coping capacity. Hence, before dissonance-reduction strategies are initiated, the individual makes an interpretation of the stimuli. Given that interpretations of stimuli likely vary both across persons and situations, the appraisal framework opens up new ways of understanding individual and situational differences in dissonance detection.

### Process Model of Emotion Regulation and Related Models

The regulatory process related to the detection of dissonance is what dissonance researchers have traditionally called the dissonance-reduction process. However, since dissonance is reduced due to the fact that it causes negative emotions, we may refer to it as an emotion-regulation process. The complex process of emotion generation and the subsequent regulatory process following an event is described by the *process model of emotion regulation* (see Gross, 2014 for a review). The process model (which is based on appraisal theories) suggests that there are five points in the emotion generative process at which individuals can regulate emotions. Each point represents a different type of regulation strategy: situation selection, situation modification, attentional deployment, cognitive change, and response modulation. To illustrate, a negative emotional event starts with a potentially emotion-arousing situation (e.g., having to make an irrevocable decision between equally attractive/unattractive options), and the first strategy individuals could employ is *avoidance* of the situation. If the situation is unavoidable, it may be possible to physically *modify* the environment in order to alter the emotional impact (e.g., ask a friend to assist you in making the tough decision). If the modification fails to regulate emotions, *deploying attention* away from the situation could work instead (e.g., after making the decision, trying to think of something else or changing the conversion topic). Certain situations, however, require a more attentive, thoughtful, and rigorous decision process (e.g., deciding which candidate to hire). In a more engaging appraisal of such a potentially dissonant situation, the individual could try to *reappraise* the situation (e.g., cognitively boosting the correctness of one’s decision or transcending the negative appraisal by trying to “see the big picture”). If the individual does not deem it fit to reappraise the situation (because it is too dissonant), or the attempt fails (due to limited cognitive resources), a full-blown emotional reaction might emerge (e.g., anger, anxiety, guilt, shame, etc.). As a last resort, the individual could modulate the response by *suppressing* these negative emotions. Note that this model assumes that an emotional regulatory response (e.g., reappraisal) might cause changes to the situation (e.g., an approach response from the individual), which in turn sets the stage for a new appraisal (e.g., more positive feelings toward the situation) and response (e.g., additional approach response). Thus, emotion regulation is a recursive dynamic process.

Based on the process model, more recent research has attempted to understand under which circumstances people choose one emotion-regulation strategy over another. Sheppes (2014) argues that emotional intensity, motivational goals (cf. Kelman and Baron, 1968), and cognitive capacity (cf. Kaplan and Crockett, 1968) will influence the decision. In a selective exposure situation with low-intensive stimuli, for instance, people will reappraise rather than distract themselves (e.g., if the headline of a news article depicts your favorite politician only slightly negatively, you might still read the article because you might reason that the content could still be interesting). The opposite pattern is observed with high-intensive stimuli. Thus, if the headline is a character-assassination of your favorite politician, you might not read the article because you suspect that the content might be too emotionally arousing. As for cognitive capacity, Sheppes argues that reappraisal (vs. distraction) is a more complex cognitive operation because it requires both attending to and elaborating on the emotional stimuli, and reappraisal might thus be avoided when the individual feels that it may be too cognitively taxing. However, Sheppes argues further that when individuals believe that they will encounter certain emotional stimuli again (i.e., stimuli related to long-term goals), they will rather reappraise the stimuli than use distraction. The opposite seems to be true for emotional stimuli encountered only once or seldomly (i.e., stimuli not related to specific goals), where distraction is more likely. Note that Sheppes’ model deals with situations in which people make more or less conscious cost-benefit analyses when deciding how to regulate emotions (see also Sheppes et al., 2011).

Recently, emotion-regulation researchers have emphasized the importance of acknowledging that a considerable amount of emotional regulatory processes occurs outside people’s awareness (Koole and Rothermund, 2011; Koole et al., 2015). In a model comparing explicit and implicit emotion regulation, Braunstein et al. (2017) organize regulation strategies along two orthogonal dimensions: the emotion regulation goal (ranging from implicit to explicit) and the emotion change process (ranging from automatic to controlled). This generates four quadrants of emotion regulation classes, where strategies range from being highly deliberate (explicit goal; controlled process) to non-deliberative (implicit goal; automatic process). Adding to the complexity, Bonanno and Burton (2013) suggest that the use of different emotion-regulation and coping strategies vary across time due to feedback regarding the efficiency of the chosen strategy, which in turn helps the individual to adjust to recurrent situations (see also Cheng, 2001; Aldao, 2013). In their view, flexibility of reduction strategies will also vary between individuals, determined by three core individual differences components: sensitivity to social context (cf. Weick, 1968), repertoire of reduction strategies, and ability to monitor feedback regarding the efficacy of the chosen strategy.

In sum, given that the purpose of dissonance reduction is to alleviate emotional tension, an emotion-regulation framework seems to be an appropriate tool for understanding the dissonance-reduction process. We suggest that the detection of dissonance might fit into the larger framework of general appraisal theories of emotion (since the detection of dissonance is the cognitive interpretation that cognitions are in conflict), and that the dissonance-reduction process could be conceptualized as emotion regulation (since dissonance-reduction strategies aim to reduce negative emotion). Next, we will explore this notion more closely by presenting a general model of dissonance reduction.

## A General Model of Dissonance Reduction

**FIGURE 1:**
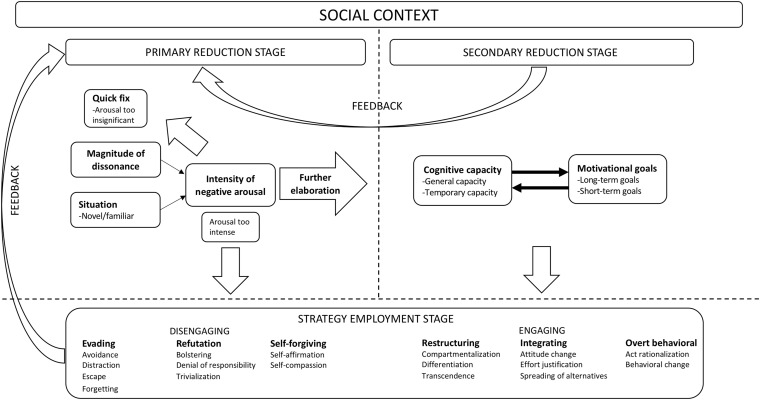
A general model of cognitive dissonance.

Applying a broader emotional perspective, we will incorporate many of the previous, seemingly disconnected, accounts of dissonance reduction into a general model (see Figure 1). This organization of past accounts will not just clarify the existing literature, it will also generate novel ideas and a new set of hypotheses not considered in past dissonance research. Note that in our model dissonance detection has already occurred, and it thus deals exclusively with the dissonance-reduction process.

### Primary Reduction Stage

In what we call the *primary reduction stage* (see left-hand side of Figure 1), the intensity of the *initial negative arousal* will be the first factor influencing how people reduce dissonance (cf. Sheppes’ model). The intensity of the initial negative arousal is in turn dependent on the *magnitude of dissonance* (i.e., all dissonant cognitions/all consonant cognitions + all dissonant cognitions), and whether the situation is *novel* or *familiar*. That is, the larger the magnitude of dissonance the more intense the negative arousal, however, novel situations may be more emotionally intense because the individual lacks an automated response to the dissonant stimuli. Note that the magnitude of dissonance and the novel-familiar dimension of the situation independently influence negative arousal in our model. For instance, every time you eat meat while trying to uphold a vegan diet produces more or less the same magnitude of dissonance, but the more often you do it the easier it might be to handle the cognitive conflict. Lastly, if the magnitude of dissonance is rather small and the situation is highly familiar, the reduction will be rather implicit (e.g., an automated distraction response). Note that the novel-familiar assessment in our model occurs at a higher-level of processing and is related to the regulatory process rather than the initial appraisal of the situation (i.e., the detection of dissonance). Similar to the process model (cf. Gross, 2014), we suggest that in the early part of the emotion-generation process, typical strategies are avoidance, escape (i.e., modification of the situation), or distraction. Furthermore, we argue that these dissonance-reduction strategies are most likely related to fear/anxiety reactions or anticipation of fear/anxiety (cf. LaBar, 2016), but also reactions related to anger (cf. Harmon-Jones and Harmon-Jones, 2016). When having enough control functions as well as sufficient motivation, however, the individual might elaborate more on the situation despite experiencing rather strong arousal. Aside from cognitive control and internal motivation, in many situations situational pressures might influence the further evaluation of dissonance stimuli. That is, we claim, in accordance with Weick’s (1968) argument, that the social context in which dissonance is evoked (e.g., presence of others vs. being alone) might dictate how people reduce dissonance.

### Secondary Reduction Stage

In the *secondary reduction stage* (see right-hand side of Figure 1), the individual has moved past the initial negative arousal and engages in more elaborate thinking about the situation. First, the individual begins to consider *motivational goals*. Some goals are long term (e.g., having a good relationship with family members), while other goals are short term (e.g., standing up for oneself in a disagreement with a stranger). When choosing to consider long-term goals in a dissonant situation, we argue, similar to Kelman and Baron (1968) and Sheppes (2014), that the individual is more likely to engage in elaborate strategies (e.g., reappraisal in the form of transcendence). When considering short-term goals in the same, however, we argue that the individual might be more likely to simply use distraction or try to escape the situation. In order to comply with long-term motivational goals, we hold that the individual will have to take *cognitive capacity* into account. Here, we distinguish between general and temporary capacity. We relate general capacity directly to the individual’s overall dissonance-reduction repertoire (cf. Kaplan and Crockett, 1968; Bonanno and Burton, 2013). For instance, some individuals have the capacity to employ sophisticated strategies (e.g., transcendence) when the situation calls for it, whereas others might only have access to more primitive strategies (e.g., trivialization or escape). Temporary capacity, in our model, refers to people’s momentary mental capacity. Sometimes people are simply too exhausted to deal with dissonant situations and might try to find the easy way out (i.e., choosing distraction, escape, or trivialization rather than transcendence, differentiation, or attitude change).

Motivational goals and cognitive capacity will of course interact across different situations. Thus, as in the primary stage, situation sensitivity is an important factor to consider in the secondary reduction stage. For instance, an individual might feel more guilt after violating a dearly held attitude, and might try harder to make amends for the violation, when this occurs in front of people that hold the same attitude (vs. in front of people that do not care about the attitude). Key to this model is also the feedback loop from motivational and cognitive interaction back to the initial interpretation and emotional reaction (i.e., from the secondary to the primary stage) Thus, motivational and cognitive factors could intensify or dampen the ongoing emotion generation process. For instance, the individual might have been too tired or simply not in the mood to deal with the dissonant situation. However, as the situation evolves the individual understands that it might be in his/her best interest to find a way to resolve the dissonant situation (see Kato, 2012, on changing coping strategies). The intensity of the emotion might increase, however, if an individual engages in a situation with the intention of trying to resolve dissonance in a constructive way but then realizes that it will not be possible. In such a scenario the consequence could be that the individual disengages (e.g., escapes, or distracts him/herself) from the situation altogether (cf. Wicklund and Brehm, 1976, on the possibility of switching dissonance-reduction strategy in the midst of using the first one, and Brehm and Cohen, 1962, on uncommitting from a dissonance situation). Thus, the dissonance-reduction process is sometimes a rather dynamic process where appraisals go back and forth several times before the individual finds a state of consonance.

Finally, in our dissonance reduction model we assume (based on the process model of emotional regulation) that any specific reduction strategy will depend on where in the regulation process the situation is located–early or late. That is, avoidance, escape, and distraction are more prevalent in situations resembling the selective exposure or free-choice paradigm (e.g., avoiding a critical article about your favorite politician, or avoiding the decision of choosing a career path in order to postpone possible post-decisional regret). However, once the individual is stuck in the situation (situations resembling induced compliance, induced hypocrisy, or effort justification) avoidance is no longer available, and distraction might be too difficult to employ. Note that in our model we assume that “further elaboration” (from primary to secondary reduction stage) could be done both explicitly and implicitly–since people might elaborate from primary to secondary reduction stage rather habitually and without much conscious effort (i.e., implicitly) over time and in recurring situations.

### Strategy Employment Stage

Once a reduction strategy is implemented, the individual’s response will feed back into the initial interpretation of the situation and a new evaluation will take place. For example, when the individual engages in a dissonant situation, a change/modification of the initial attitude might take place. Interestingly, this may in turn lead to more positive emotions toward the situation–since the individual has managed to accommodate the modified attitude into the existing cognitive structure. Empirical findings, across several experiments, have actually shown a strong positive relationship between positive emotions and attitude change in the induced-compliance paradigm (Cancino-Montecinos et al., 2018). Thus, the recursive nature of the dissonance-reduction process alters the subsequent emotional experience in relation to the dissonant situation–which depends on how the individual reduces dissonance in that particular situation (see next section).

We have organized reduction strategies into two broad categories: *Engaging* and *Disengaging* (cf. categorization of coping strategies, Carver and Connor-Smith, 2010). The general idea is that disengaging strategies (avoidance, escape, trivialization, etc.) occur earlier in the dissonance-reduction process and are less cognitively taxing, whereas engaging strategies (transcendence, attitude change, spreading of alternatives, etc.) occur later in the process after more cognitive elaboration–although people could disengage after an initial elaboration of the situation. Furthermore, both categories of strategies are further classified into subcategories. Disengaging strategies such as avoidance and escape are called *evading* strategies, since those strategies do not confront the situation head-on and require the least amount of effort. Strategies such as trivialization and bolstering are called *refutation* strategies and are more effortful since they require more cognitive elaboration of the stimuli, and they are characterized by an explicit non-acceptance of the dissonant cognitions. The third subtype of the disengaging category is called *self-forgiving* strategies and includes strategies such as self-affirmation and self-compassion. This subtype is mainly characterized by reducing the negative emotions through cognitively highlighting unrelated positive aspects of one’s persona–rather than engaging with the dissonant situation. Engaging strategies such as transcendence and differentiation are called *restructuring* strategies, since their aim is simply to rearrange the cognitions (both dissonant and consonant) and create a new structure (or several new structures). Other engaging strategies, such as attitude change and spreading of alternatives, aim to incorporate dissonant cognitions into the existing consonant structure and are therefore called *integrating* strategies. Lastly, act rationalization and behavior change are simply called *overt behavioral* strategies, since these have an explicit action component whereby an individual reduces dissonance.

We argue that the quick fix route in the primary reduction stage is an implicit-automated response, since it occurs before any deeper elaboration of the dissonant stimuli (cf. Braunstein et al., 2017). As for the habitual responses at this stage, habitual avoidance of certain dissonant information may be a more implicit process than trivialization, since trivialization involves the downplaying of cognitions. In the secondary evaluation stage, any automated goal pursuit should be more implicit than cost-benefit analyses performed via the conscious consideration of motivational goals and cognitive capacity. Given this, cognitive reappraisal is considered to be the most explicit type of emotion regulation (cf. Braunstein et al., 2017).

## Predicting Reduction Strategies: Some Ideas

Throughout this paper, we make the point that dissonance reduction is a form of emotion regulation. The purpose is to provide an encompassing model that also highlights both the role of specific emotions (or clusters of emotions) and the role of individual differences in emotion regulation in order to predict how people might reduce dissonance.

Elliot and Devine (1994) were the first to investigate more closely the discrete emotions in relation to a dissonant situation. They found that affective states such as *uncomfortable*, *uneasy*, and *bothered* (i.e., general discomfort) were related to the attitude-change effect in the induced-compliance paradigm. In a more recent simulation study, Kenworthy et al. (2011) found that the emotion *guilt* was most clearly associated with the outcome variable across paradigms. Since there is great variability in how people interpret a dissonant situation, and then choose to reduce dissonance, we believe that there might be more than *one* discrete emotion (or affective state) associated with the dissonance-reduction process. In other words, we argue for a more pluralistic view of the dissonance-reduction process (see Table 2).

**TABLE 2:** Specific emotions associated with specific reduction strategies.

Emotion experienced during dissonance	Dissonance-reduction strategy	Emotional outcome after success
Anger	Attitude bolstering	Satisfaction
Irritation	Denial of responsibility	Relief
Anxiety/Fear	Avoidance Denial of information Forgetting	Relief
Guilt	Behavioral change	Serenity
Shame	Distraction Escape	Sadness

If the individual experiences high-arousal negative emotions such as anger, hostility, and/or frustration in a dissonant situation (e.g., after being asked to write a counter-attitudinal essay in the induced-compliance paradigm), attitude bolstering might be the most likely reduction strategy. Since these emotions are approach motivated, the individual will try to quickly find the external source of the emotions and extinguish this. Since the model is recursive, the subsequent emotion might be a feeling of satisfaction, for example when successfully standing up for oneself when someone (parent, spouse, boss, etc.) has induced you to behave in a way that contradicts your attitude/values. However, when such attempts to stand up for oneself in anger-dominated situations fail, the anger might be replaced by a depressed mood and avoidance behavior instead (see, e.g., Harmon-Jones and Harmon-Jones, 2016). If the feeling is slight irritation, rather than full-blown anger, the individual might be fine with simply denying responsibility for the dissonant behavior (i.e., finding an external cause for the behavior). If successfully implemented, the individual might feel relief after denying responsibility. However, if the reduction strategy fails, the individual might feel a prolonged sense of annoyance and irritation.

If the individual’s experience is dominated by fear and anxiety (also high-arousal negative emotions), avoidance, escape, or distraction are likely dissonance-reduction strategies. Since fear and anxiety are avoidance motivated emotions, these should be typical types of responses (LaBar, 2016). If the dissonance-reduction strategy is implemented successfully, the subsequent emotional experience will likely be characterized by relief (e.g., after avoiding an irrevocable decision). However, if the individual does *not* manage to fully execute the reduction attempt, rumination and counter-factual thinking may ensue, or prolonged feelings of distress and uneasiness–especially if anxiety dominated the emotional experience (see, e.g., Gratz and Roemer, 2004; Watkins, 2004; Nolen-Hoeksema et al., 2008;, on difficulties in emotion regulation). Interestingly, Festinger (1957) himself entertained the idea that some individuals might be so overwhelmed by the dissonance arousal that they would have difficulties in finding a proper reduction strategy.

As for guilt (a lower-intensity self-conscious emotion), this emotion is experienced when people acknowledge having violated standards, rules and/or goals (SRGs) (Lewis, 2016). For example, using a car when walking is completely feasible or not giving up one’s seat for an elderly individual on the subway might produce feelings of guilt. Since guilt usually has a corrective response, the individual might try to make amends for the transgressions in these cases (e.g., behavioral change). The subsequent emotion (after “making up” for bad behavior) might be a feeling of serenity. However, a failed attempt to make up might lead to continued guilt, which might in turn lead to the individual trying to compensate with an indirect gesture rather than addressing the main issue.

Shame is another self-conscious emotion likely to emerge in dissonant situations related to violations of SRGs–particularly in situations where there is not much the individual can do to compensate for the dissonant behavior, and when the individual attributes the violation of SRGs to the global self. Here, the individual is more likely to retreat from, rather than approach, the situation (Lewis, 2016). Thus, escape or distraction (after the feedback loop from the secondary to the primary evaluation stage) might be the likely reduction strategies employed in these situations. Note that shame could linger for some time, meaning that it might be difficult to experience immediate relief after the situation (Lewis, 2016). In the worst cases, it could lead to a prolonged sense of sadness.

As for reduction strategies related to the categories of restructuring (e.g., transcendence, compartmentalization) and integration (e.g., effort justification, spreading alternatives), they are not related to full-blown emotions–but are possibly related to affect-like discomfort (cf. Elliot and Devine, 1994) lingering from the initial interpretation of the situation (i.e., primary evaluation stage). Since these strategies imply that the individual managed to somehow resolve the situation, a full-blown negative emotion is unlikely to have evolved (or is at least unlikely to still be present). After employing integrating strategies, the resulting emotional experience might be feelings of excitement (e.g., after increasing the degree of liking a valued goal) and/or optimism (e.g., after making a tough decision) (cf. Cancino-Montecinos et al., 2018 on positive emotions related to attitude change). As for restructuring strategies, the individual will likely be at peace and experience a feeling of content and relaxation after employing strategies that manage to resolve dissonance without having to fundamentally change their cognitive structure.

As discussed above, there is good reason to suggest that some clusters of emotions might be related to specific reduction strategies. Note, however, that these suggestions are not part of the core assumptions of the general model.

## Feasibility of the Emotion Regulation Perspective on Dissonance-Reduction Processes

A central assumption of this general model is the pluralistic view on emotional reactions to cognitive dissonance and the subsequent reduction process. In other words, people can react vastly different to the same dissonant situation and then resolve the situation in several different ways. If a broader emotion-regulation conceptualization of the dissonance-reduction process is of any use at all, any given dissonance experiment should certainly provide some hints. Since this conceptualization assumes that any given situation can give rise to a multitude of interpretations, emotional reactions, and dissonance-reduction attempts, people’s potential variation of emotional reactions to the same dissonant situation should be an obvious indication of the usefulness. Another obvious indication of the usefulness would be the detection of several different dissonance-reduction strategies in the same situation. In this section, we discuss and illustrate how methodological decisions (e.g., experimental set-up and data-analyses) can influence which theoretical conclusions researchers draw from dissonance studies.

### Past Methodological Issues

In general, one problematic feature of past dissonance studies is the employment of between-group designs (experiment group vs. control group), were individuals are treated as homogenous entities reacting more or less identically to the manipulation. Any deviation from the expected reaction within the experiment group is treated as error variance. Consequently, theoretical conclusions are then based on mean-score differences between groups–regardless of how large the overlap between score distributions, as long as the difference is statistically significant at alpha level 0.05. Given this common practice, studies have rarely pre-measured people’s initial attitudes, and/or the perceived importance of that attitude (which is supposed to decrease if one trivializes) (i.e., in a within-subjects design). However, without pre-measures it is impossible to know whether individuals have moved up or down an attitude scale or attitude-importance scale (in relation to their initial position) during the experiment. Additionally, past researchers have seldom measured people’s self-reported emotional reactions to the dissonant situation. Those few who have done so, have asked individuals to state what they feel “at the moment,” and not what they feel in relation to the dissonant situation (i.e., “to what extent did you experience these emotions while writing the essay?”). The former formulation could give rise to substantial measurement error, since individuals might report current mood or general emotionality rather than emotions related to the situation (see Cancino-Montecinos et al., 2018 for an empirical study on this point). Further, studies have focused on single emotions (e.g., guilt) or affective states (e.g., discomfort) in relation to a certain reduction strategy (predominantly attitude change). Since we argue that any given situation can give rise to a multitude of interpretations, emotional reactions, and dissonance-reduction attempts, a proper understanding of the dissonance-reduction process requires the investigation of how a *single* individual reacts from one point in time to another–not how one group (experimental group) versus another group (control group) reacts at a single point in time. Moreover, it requires the investigation of how (and to what extent) the individual feels emotionally *during the dissonant situation*, and not how the individual feels in general. Thus, by assessing the *prevalence* of different emotional reactions and different dissonance-reduction strategies in the same situation, researchers are able to tap into the multifaceted nature of dissonance reduction, and thereby discover the usefulness of an emotion-regulation perspective.

### Remedies for Past Methodological Shortcomings: A Practical Example

As stated above, a key feature of emotion-regulation is that, in any given situation, people might differ quite dramatically in their interpretation, emotional reaction, and their subsequent regulatory attempt. Thus, including a measurement of a wider variety of emotional and affective reactions in a typical dissonance experiment (e.g., induced compliance) makes it possible to assess to what extent individuals experience, for instance, more anger-like, anxiety/fear-like, or self-conscious-like emotions, or *overall* negative emotions. Additionally, one can assess to what extent people experienced both positive *and* negative emotions, or very low levels of emotions overall. A straightforward way to group individuals into these categories is to first investigate via factor analysis whether different patterns emerge (e.g., an anger and anxiety/fear factor), and then simply count how many individuals experienced, for instance, predominately more anger-like emotions (vs. other emotions) during the manipulation. As for dissonance-reduction strategies, with the attitude and importance of attitude pre-measures (preferably measured several days in advance in order to avoid having these measures highly accessible at the time of the manipulation), one can ascertain whether an individual (1) changed attitude, (2) maintained the original attitude, or (3) strengthened the original attitude (attitude bolstering). One can also ascertain whether the individual (1) decreased the importance of the attitude (trivialize), (2) maintained the original importance of the attitude, or (3) strengthened the importance of the attitude (importance bolstering). Thus, with regard to the initial attitude and the attitude importance, the potential outcomes can be captured within a 3 × 3 matrix: (1) Attitude change only; (2) Attitude bolstering only; (3) Trivialization only; (4) Importance bolstering only; (5) Attitude change and Trivialization; (6) Attitude change and Importance bolstering; (7) Attitude bolstering and Trivialization; (8) Attitude bolstering and Importance bolstering; and (9) No strategy.

### Demonstration of Feasibility

For the purpose of illustrating how the choice of experimental set-up and data analysis affects results, and thereby possible conclusions about theory, data from a within-subjects induced-compliance study (similar to the one described in the practical example; Cancino-Montecinos et al., 2018) was re-analyzed in terms of prevalence of emotional reactions and reduction strategies. Note that this demonstration is simply meant for the above-mentioned purpose (and thereby illustrating the feasibility of the emotion-regulation perspective)–not as a test of the model presented earlier. Such an attempt would require a multitude of studies investigating participants within and across several different dissonant situations, with an array of different situational manipulations, as well as controlling for individual differences–and perhaps including a longitudinal study (see future directions section).

In short, participants in this experiment were asked a week in advance to state their attitude, and the importance of that attitude, on different university related issues (the target issue being a possible reduction of students’ financial aid when failing exams). When arriving at the lab (a week later), they were asked if they wished to participate in a university survey where they had to argue (by writing a short essay) for the reduction of students’ financial aid. After completing the essay, they were asked to state to what extent they had experienced positive and negative emotions while writing the essay. Lastly, they were asked to state (as the week prior) their attitude, and the importance of the attitude, toward a possible reduction of students’ financial aid. The dataset for the analyses can be found at: osf.io/z5sy6. Note that this study was conducted in accordance with the ethical principles outlined in European Code of conduct for Research Integrity Revised version (ALLEA–All European Academies, 2017). The study did not include factors that require ethical vetting according to Swedish legislation on research ethics, the act concerning the ethical review of research involving humans, SFS (2003). This was also confirmed by the head of Department of Psychology at Stockholm university (see Cancino-Montecinos et al., 2018, for more details).

### Prevalence and Variation of Emotional Reactions

The analysis revealed that for some individuals, the emotional experience of the dissonant situation was predominantly characterized by either Anger, Anxiety/Fear or Self-consciousness. Other individuals experienced overall negative or overall positive emotions, whereas a considerable portion (almost 20 percent) experienced a mixture of both positive and negative emotions. Lastly, some individuals experienced very low overall levels of emotions (see Table 3). Thus, focusing simply on the total mean score would conduce to misrepresentation of how individuals experienced the dissonant situation. The dissonance experience does not seem to be characterized by *one* specific emotion, affective state, or pattern of emotions–since people vary in their interpretation and subsequent emotional reaction.

**TABLE 3:** Mean (and standard deviation) for different negative emotion factors, all negative emotions, and all positive emotions in the different groups.

	Anger factor	Anxiety/Fear factor	Self-conscious factor	All negative emotions	All positive emotions
Anger-dominant (*n* = 18)	3.83 (1.57)	0.96 (1.17)	0.61 (0.87)	2.21 (1.12)	0.83 (0.76)
Anxiety/Fear-dominant (*n* = 8)	3.00 (1.21)	4.25 (1.33)	0.56 (0.56)	2.90 (0.93)	1.33 (1.14)
Self-conscious dominant (*n* = 7)	1.86 (1.21)	0.95 (0.97)	3.29 (1.04)	1.92 (0.81)	0.93 (0.77)
Overall negative (*n* = 24)	3.97 (1.41)	3.44 (1.31)	2.85 (1.77)	3.41 (1.00)	0.94 (0.55)
Overall positive (*n* = 14)	0.70 (0.75)	0.93 (0.83)	0.82 (0.97)	0.79 (0.64)	3.38 (1.04)
Mixed emotions (*n* = 20)	2.88 (1.16)	1.60 (1.57)	1.88 (1.51)	2.20 (0.97)	2.95 (1.01)
Overall low (*n* = 15)	0.60 (0.67)	0.51 (0.49)	0.37 (0.52)	0.50 (0.36)	0.81 (0.62)
TOTAL (*n* = 106)	2.27 (1.79)	1.82 (1.71)	1.52 (1.60)	2.08 (1.34)	1.63 (1.32)

*Scale ranged from 0 to 7. Factor analysis revealed three factors for negative emotions and one factor for positive emotions. Discomfort and Sadness are not included in these factors due to indistinct loadings. Anger factor (Anger, Frustration, Hostility, Irritation); Anxiety/Fear factor (Anxiety, Fear, Nervousness) Self-conscious factor (Guilt, Shame); All negative emotions (Anger, Frustration, Hostility, Irritation, Anxiety, Fear, Nervousness, Guilt, Shame, Discomfort, Sadness); All positive emotions (Enthusiasm, Excitement, Hope, Inspiration, Joy, Kindness, Optimism, Pride, Relief, Strength).*

### Prevalence and Variation of Dissonance-Reduction Strategies

Furthermore, the analysis reveals that all possible outcomes from the 3 × 3 matrix were represented (see Table 4). Interestingly, a considerable portion (almost one-fourth) changed attitude *and* trivialized in the same dissonant situation–two strategies that have been regarded as mutually exclusive by some researchers (e.g., Simon et al., 1995). As with varying emotional reactions, people seem to differ substantially regarding how they resolve cognitive dissonance within the same situation. Thus, focusing only on total mean scores is a clear misrepresentation in this case as well.

**TABLE 4:** Mean (and standard deviation) for pre- and post-attitude, and pre- and post-importance of attitude in the different dissonance-reduction strategies.

Dissonance-reduction strategy	Pre-attitude	Post-attitude	Pre-importance of attitude	Post-importance of attitude
1. Attitude change (*n* = 9)	0.89 (0.60)	3.00 (1.23)	3.67 (1.80)	3.67 (1.80)
2. Attitude bolstering (*n* = 1)	3	1	6	6
3. Trivialization (*n* = 34)	0.24 (0.55)	0.24 (0.55)	6.24 (1.23)	3.26 (2.23)
4. Importance bolstering (*n* = 11)	0.18 (0.60)	0.18 (0.60)	3.82 (1.89)	5.55 (1.51)
5. Attitude change and Trivialization (*n* = 25)	0.64 (0.95)	2.92 (1.71)	5.44 (1.16)	2.80 (1.58)
6. Attitude change and Importance bolstering (*n* = 7)	1.14 (1.46)	3.14 (2.12)	3.43 (1.90)	5.00 (1.73)
7. Attitude bolstering and Trivialization (*n* = 3)	1.33 (0.58)	0.33 (0.58)	4.00 (1.73)	1.67 (2.89)
8. Attitude bolstering and Importance bolstering (*n* = 2)	2.50 (0.71)	0.00 (0.00)	2.50 (0.71)	5.50 (2.12)
9. No strategy (*n* = 14)	0.50 (0.94)	0.50 (0.94)	6.07 (1.07)	6.07 (1.07)
TOTAL (*n* = 106)	0.58 (0.92)	1.33 (1.74)	5.24 (1.74)	3.93 (2.18)

*Scale ranged from 0 to 7.*

In sum, these simple re-analyses demonstrate the danger in over-emphasizing general trends in typical dissonance studies. They also show that conceptualizing dissonance reduction in the broader terms of emotion-regulation could be a viable approach moving forward.

## General Discussion

Our aim was to present a general model of dissonance-reduction that would transcend specific experimental paradigms, reduction strategies, and moderators, and thereby generate new theoretical ideas and testable hypotheses. This was achieved by applying a broader emotional perspective, coupled with some empirical demonstrations. Variability across situations and individuals (cf. Mischel and Shoda, 1995; Fleeson, 2004) is one of the central ideas generated from our model. Related to this, flexibility in the use of reduction strategies and change over time, and feedback loops enabling new interpretations of the dissonant situation, are further novel concepts generated from our model. Some novel predictions are that (a) the dissonance-reduction process is more multifaceted (both emotionally and cognitively) than previous accounts have suggested; (b) some individuals will learn from (or adapt to) the social environment more than others, and this will lead to more flexible use of reduction strategies over time and across situations; and (c) specific emotions (or clusters of emotions) experienced during the dissonant situation might be related to specific reduction strategies. Furthermore, our emotional perspective also led to a novel categorization of dissonance-reduction strategies.

It is important to point out that the model presented here is a working model, and therefore some details are less clear than others. The distinction between explicit and implicit dissonance reduction needs to be further elaborated. For instance, engaging strategies (e.g., attitude change, spreading of alternatives, effort justification) might not be particularly conscious. That is, although attitude change is an approach-related action (Harmon-Jones et al., 2011) which leads to more positive emotions (Cancino-Montecinos et al., 2018), the individual might not be aware of the chosen strategy. Furthermore, given the dynamic nature of the human mind, any excessive use of an explicit reduction strategy might render it more implicit over time. Braunstein et al. (2017) also present some strategies as inherently implicit, such as extinction and reinforcer revaluation, where experience-based learning enables the individual to update mental schemas about the value of certain stimuli that might have changed (from negative to neutral or positive). Thus, much of people’s everyday dissonance reduction could be occurring via implicit adaptation rather than conscious effortful evaluations. As we have stated above, elaboration of dissonant stimuli (from primary to secondary reduction stage) could be done both explicitly and implicitly.

Although we argue that motivational goals and cognitive capacity are key to understanding the secondary evaluating process, there are perhaps factors such as self-efficacy in emotion regulation (Caprara et al., 2008), or the persons’ own implicit theories about their emotion regulation (Tamir et al., 2007; Kappes and Schikowski, 2013) which could play a vital role in this process. That is, people might not engage in more effortful emotion regulation (e.g., reappraisal) due to a lack of belief in their ability, or because they believe that emotions are fixed entities.

Another challenging issue is the categorization of reduction strategies, which has also been a notoriously difficult task in the coping literature (Skinner et al., 2003). The empirical data clearly shows that, for instance, many individuals used attitude change and trivialization (see Webb et al., 2012, on the simultaneous use of different emotion-regulation strategies). A possible explanation is perhaps that trivialization coupled with attitude change is qualitatively different from trivialization alone. This type of trivialization might actually assist the attitude-change process in a rather complex cognitive reappraisal procedure.

### Toward a Pluralistic Approach

There is a need for a more pluralistic approach to the investigation of dissonance reduction, since the reduction process is a multi-layered phenomenon that could be studied across the spectrum of intra- and inter-psychological space, and across the space of weak and strong situations. For instance, to date there has not been any large-scale attempt to examine the role of individual difference in dissonance reduction. Although research has provided some important insights into how personality can moderate dissonance reduction, the findings are rather disconnected from each other and somewhat ambiguous (e.g., both high and low self-esteem have been related to attitude change), and they have focused almost exclusively on a single outcome variable (i.e., attitude change) (see Abelson et al., 1968; Wicklund and Brehm, 1976; Harmon-Jones et al., 2009; for more extensive reviews on individual differences in dissonance research). A serious take on an individual difference approach requires assessments of the individual’s response to cognitive dissonance across different experimental paradigms (free choice, induced compliance, effort justification, etc.), and testing a multitude of variables in order to disentangle what is common and what is unique to specific situations (and to specific reduction strategies). Since we argue that the dissonance-reduction process should be regarded as emotion regulation, individual differences in *reactivity* (biologically based reactions to changes in the external and internal environment; Rothbart et al., 2014), *effortful control* (self-control processes consistently monitoring and regulating reactivity; Rueda, 2012), and *emotional competence* (awareness of one’s own and others’ emotional states, acceptance of one’s emotions and being confident in expressing these, as well as coping with aversive or distressing emotions; Saarni, 1999) might shed some light here. A possible prediction would be that individuals high on reactivity and low on effortful control (measured with the adult temperament questionnaire; Evans and Rothbart, 2007) will go straight for the habitual response while others (thanks to effortful control) might be able to withstand emotional turmoil and evaluate the dissonant situation more thoroughly. As for emotional competence, one could predict that an emotionally competent (measured with the trait-meta-mood scale; Salovey et al., 1995) individual would be more aware of his/her own hypocrisy, might be able to accept the reality of the situation and maybe choose to “just let it go” or try to “be a better person from now on”–instead of denying responsibility or trivializing the behavior.

### Direction for Future Research

Aside from the individual differences approach, future dissonance research should involve a broader set of methodological approaches to the study of dissonance reduction. Longitudinal designs, experience sampling, multi-trait analyses, non-linear analyses, and more qualitative analyses will undoubtedly advance our understanding of the dissonance-reduction process. For instance, from a lifespan perspective it is obvious that an individual will probably not reduce dissonance in the same way at 55 years of age vs. 21 years of age (e.g., middle aged and older individuals are less prone to engage in aversive situations). Given that most research on dissonance has been performed on university students, a lifespan perspective might be a very important issue to consider. As for qualitative analyses, using a think aloud protocol in typical dissonant experiments might give researchers more insight into people’s thought process during the dissonant episode. Another way to approach people’s thoughts might be to ask them to indicate *why* (in one or a couple of sentences) they experience certain emotions during a dissonant situation. Furthermore, a non-linear perspective could also propel dissonance theory forward. From this viewpoint, people’s predisposed actions, thoughts, and emotions are inherently dynamic, indicating that constant change (due to internal mechanisms and external forces) is the true flow of human psychology (see e.g., Nowak et al., 2005; Guastello et al., 2008; Read and Simon, 2012; Vallacher et al., 2015). In a dissonance context, this could explain how, over time, feedback from the social environment alters the individual’s thoughts and emotions in different situations–eventually changing habitual responses and giving rise to new ways of reducing dissonance. Based on a Brunswikian approach (Brunswik, 1955), another possible suggestion for future research is to map the universe of different dissonant situations–that is, creating a taxonomy of dissonant situations (cf. the DIAMONDS taxonomy of major dimensions of situation characteristics; Rauthmann et al., 2014). In this way it would be easier for researchers to both understand the nature of specific situations, and the nature of overarching dissonance structures. Further, thinking about the universe of dissonant situations and dissonance-reduction strategies could help to understand how these concepts relate to other areas of psychological inquiry, as well as the boundary conditions for these concepts. Thus, this approach may contribute to the empirical study of dissonance research. Finally, on a more general note, our model could also help understand how people cope with more distressing life events. That is, how people tend to reduce dissonance might actually be an important hint as to how people handle major life events. In a similar vein, since our model includes both traditional emotion-regulation strategies (avoidance, distraction, cognitive reappraisal) and more traditional coping strategies (behavioral change, act rationalization), it could also help bridge the gap between emotion regulation and coping mechanisms.

In sum, future exploration should focus on questions such as (a) what is general about dissonance reduction, (b) what is specific to certain situations, (c) what is specific to certain individuals, (d) how do individuals vary from situation to situation (and over time), and (e) how do people reduce dissonance outside of the laboratory context.

## Final Remarks

Our theoretical contribution reconciles past ideas regarding dissonance reduction by simply commencing from the core (emotional) premise of dissonance theory (i.e., cognitive dissonance causes negative emotions which the individual will be motivated to reduce, and similar situations will probably be avoided in the future). In essence, the present theoretical paper demonstrates that previous accounts of how people use dissonance-reduction strategies are bound to specific cases and methodological constraints. We therefore proposed a (working) dissonance-reduction model that transcends specific experimental paradigms and reduction strategies. Specifically, we found that a wider theoretical perspective and a more pluralistic approach to research design results in a richer understanding of the psychological phenomena of dissonance reduction. Applying the suggested emotion-regulation framework on dissonance research may hopefully open up new avenues of inquiry and help bring dissonance theory into the second decade of the twenty-first century.

## Data Availability Statement

The dataset for this paper is available at osf.io/z5sy6.

## Ethics Statement

The studies involving human participants were reviewed and approved by Håkan Fischer, Department of Psychology at Stockholm University. The patients/participants provided their written informed consent to participate in this study.

## Author Contributions

SC-M reviewed the literature, wrote the draft, and performed the analyses. All authors developed the theoretical framework and wrote the final manuscript.

## Conflict of Interest

The authors declare that the research was conducted in the absence of any commercial or financial relationships that could be construed as a potential conflict of interest.

## References

[B1] Abelson R. P. (1959). Modes of resolution of belief dilemmas. *J. Conf. Reso.* 3 343–352. 10.1177/002200275900300403

[B2] Abelson R. P. Aronson E. McGuire W. J. Newcombe T. M. Rosenberg M. J. Tannenbaum P. H. (1968). *Theories of Cognitive Consistency: A Sourcebook.* Chicago, IL: Rand-McNally.

[B3] Aldao A. (2013). The future of emotion regulation research: capturing context. *Perspec. Psychol. Sci.* 8 155–172. 10.1177/1745691612459518 26172497

[B4] ALLEA–All European Academies (2017). *European Code of Conduct for RESEARCH Integrity Revised Version.* Berlin: ALLEA, All European Universities.

[B5] Aronson E. (1969). “The theory of cognitive dissonance: a current perspective,” in *Adv. Exp. Soc. Psychol.*, *4*, ed. Berkowitz L. (New York, NY: Academic Press), 2–34. 10.1016/S0065-2601(08)60075-1

[B6] Aronson E. (1992). The return of the repressed: dissonance theory makes a comeback. *Psychol. Inquiry* 3 303–311. 10.1207/s15327965pli0304_1

[B7] Aronson E. (1999). “Dissonance, hypocrisy, and the self-concept,” in *Cognitive Dissonance: Progress on a Pivotal Theory in Social Psychology*, eds Harmon-Jones E. Mills J. (Washington, DC: American Psychological Association), 103–126. 10.1037/10318-005

[B8] Aronson E. Mills J. (1959). The effect of severity of initiation on liking for a group. *J. Abnorm. Soc. Psychol.* 59 177–181. 10.1037/h0047195

[B9] Aronson J. Cohen G. Nail P. R. (2019). “Self-affirmation theory: An update and appraisal,” in *Cognitive Dissonance: Reexamining a Pivotal Theory in Psychology*, ed. Harmon-Jones E. (Washington, DC: American Psychological Association), 159–174. 10.1037/0000135-008

[B10] Beauvois J. L. Joule R. V. (1996). *A Radical Dissonance Theory.* London: Taylor & Francis.

[B11] Beauvois J. L. Joule R. V. (1999). “A radical point of view on dissonance theory,” in *Cognitive Dissonance: Progress on a Pivotal Theory in Social Psychology*, eds Harmon-Jones E. Mills J. (Washington, DC: American Psychological Association), 43–70. 10.1037/10318-003

[B12] Beauvois J. L. Joule R. V. Brunetti F. (1993). Cognitive rationalization and act rationalization in an escalation of commitment. *Bas. Appl. Soc. Psychol.* 14 1–17. 10.1207/s15324834basp1401_1

[B13] Bonanno G. A. Burton C. L. (2013). Regulatory flexibility: an individual differences perspective on coping and emotion regulation. *Perspec. Psychol. Sci.* 8 591–612. 10.1177/1745691613504116 26173226

[B14] Braunstein L. M. Gross J. J. Ochsner K. N. (2017). Explicit and implicit emotion regulation: a multi-level framework. *Soc. Cog. Aff. Neurosci.* 12 1545–1557. 10.1093/scan/nsx096 28981910 PMC5647798

[B15] Brehm J. W. (1956). Postdecision changes in the desirability of alternatives. *J. Abnorm. Soc. Psychol.* 52 384–389. 10.1037/h0041006 13318848

[B16] Brehm J. W. Cohen A. R. (1962). *Explorations in Cognitive Dissonance.* New York, NY: Wiley 10.1037/11622-000

[B17] Brunswik E. (1955). Representative design and probabilistic theory in a functional psychology. *Psychol. Rev.* 62:193. 10.1037/h0047470 14371898

[B18] Cancino-Montecinos S. Björklund F. Lindholm T. (2018). Dissonance reduction as emotion regulation: attitude change is related to positive emotions in the induced compliance paradigm. *PLoS One* 13:e0209012. 10.1371/journal.pone.0209012 30557326 PMC6296533

[B19] Caprara G. V. Di Giunta L. Eisenberg N. Gerbino M. Pastorelli C. Tramontano C. (2008). Assessing regulatory emotional self-efficacy in three countries. *Psychol. Assess.* 20 227–237. 10.1037/1040-3590.20.3.227 18778159 PMC2713723

[B20] Carver C. S. Connor-Smith J. (2010). Personality and coping. *Annu. Rev. Psychol.* 61 679–704. 10.1146/annurev.psych.093008.100352 19572784

[B21] Cheng C. (2001). Assessing coping flexibility in real-life and laboratory settings: a multimethod approach. *J. Pers. Soc. Psychol.* 80 814–833. 10.1037/0022-3514.80.5.814 11374752

[B22] Cooper J. (2007). *Cognitive Dissonance: Fifty Years of a Classic Theory.* Thousand Oakes, CA: Sage.

[B23] Cooper J. (2019). “In Search of the motivation for dissonance reduction: the drive to lessen aversive consequences,” in *Cognitive Dissonance: Reexamining a Pivotal Theory in Psychology*, ed. Harmon-Jones E. (Washington, DC: American Psychological Association), 175–193. 10.1037/0000135-009

[B24] Cooper J. Fazio R. H. (1984). “A new look at dissonance theory,” in *Adv. Exp. Soc. Psychol.*, *17*, ed. Berkowitz L. (Orlando, FL: Academic Press), 229–266. 10.1016/s0065-2601(08)60121-5

[B25] Devine P. G. Tauer J. M. Barron K. E. Elliot A. J. Vance K. M. (1999). “Moving beyond attitude change in the study of dissonance-related processes,” in *Cognitive Dissonance: Progress on a Pivotal Theory in Social Psychology*, eds Harmon-Jones E. Mills J. (Washington, DC: American Psychological Association), 297–323. 10.1037/10318-012

[B26] Elliot A. Devine P. (1994). On the motivational nature of cognitive dissonance: dissonance as psychological discomfort. *J. Pers. Soc. Psychol.* 67 382–394. 10.1037/0022-3514.67.3.382

[B27] Evans D. E. Rothbart M. K. (2007). Developing a model for adult temperament. *J. Res. Pers.* 41 868–888. 10.1016/j.jrp.2006.11.002

[B28] Festinger L. (1957). *A Theory of Cognitive Dissonance.* Stanford, CA: Stanford University Press.

[B29] Festinger L. Carlsmith J. M. (1959). Cognitive consequences of forced compliance. *J. Abnorm. Soc. Psychol.* 58 203–210. 10.1037/h0041593 13640824

[B30] Fleeson W. (2004). Moving personality beyond the person-situation debate: The challenge and the opportunity of within-person variability. *Curr. Direc. Psychol. Sci.* 13 83–87. 10.1111/j.0963-7214.2004.00280.x

[B31] Gawronski B. Ye Y. Rydell R. J. De Houwer J. (2014). Formation, representation, and activation of contextualized attitudes. *J. Exp. Soc. Psychol.* 54 188–203. 10.1016/j.jesp.2014.05.010

[B32] Gosling P. Denizeau M. Oberlé D. (2006). Denial of responsibility: a new mode of dissonance reduction. *J. Pers. Soc. Psychol.* 90 722–733. 10.1037/0022-3514.90.5.722 16737370

[B33] Gratz K. L. Roemer L. (2004). Multidimensional assessment of emotion regulation and dysregulation: Development, factor structure, and initial validation of the difficulties in emotion regulation scale. *J. Psychopat. Behav. Asses.* 26 41–54. 10.1023/b:joba.0000007455.08539.94

[B34] Gross J. J. (2014). “Emotion regulation: conceptual and empirical foundation,” in *Handbook of Emotion Regulation*, 2nd Edn, ed. Gross J. J. (New York, NY: Guilford Press), 3–20.

[B35] Guastello S. J. Koopmans M. Pincus D. (eds.) (2008). *Chaos and Complexity in Psychology: The Theory of Nonlinear Dynamical Systems.* Cambridge: Cambridge University Press 10.1017/CBO9781139058544

[B36] Hardyck J. A. Kardush M. (1968). “A modest modish model for dissonance reduction,” in *Theories of Cognitive Consistency: A Sourcebook*, eds Abelson R. P. Aronson E. McGuire W. T. Newcomb T. M. Rosenberg M. J. Tannenbaum P. H. (Chicago, IL: Rand-McNally), 684–692.

[B37] Harmon-Jones C. Schmeichel B. J. Inzlicht M. Harmon- Jones E. (2011). Trait approach motivation relates to dissonance reduction. *Soc. Psychol. Pers. Sci.* 2 21–28. 10.1177/1948550610379425

[B38] Harmon-Jones E. Amodio D. M. Harmon-Jones C. (2009). “Action-based model of dissonance: a review, integration, and expansion of conceptions of cognitive conflict,” in *Adv. Exp. Soc. Psychol*, Vol. 4 ed. Zanna M. (Cambridge, MA: Elsevier Academic Press), 119–166. 10.1016/S0065-2601(08)00403-6

[B39] Harmon-Jones E. Harmon-Jones C. (2016). “Anger,” in *Handbook of Emotions*, 4th Edn, eds Barrett L. F. Lewis M. Haviland-Jones J. M. (New York, NY: Guildford Press), 774–791.

[B40] Kaplan B. Crockett W. H. (1968). “Developmental analysis of modes of resolution,” in *Theories of Cognitive Consistency: A Sourcebook*, eds Abelson R. P. Aronson E. McGuire W. T. Newcomb T. M. Rosenberg M. J. Tannenbaum P. H. (Chicago, IL: Rand−McNally), 661–669.

[B41] Kappes A. Schikowski A. (2013). Implicit theories of emotion shape regulation of negative affect. *Cogn. Emot.* 27 952–960. 10.1080/02699931.2012.753415 23282147

[B42] Kato T. (2012). Development of the coping flexibility scale: evidence for the coping flexibility hypothesis. *J. Coun. Psychol.* 59 262–273. 10.1037/a0027770 22506909

[B43] Kelman H. C. Baron R. M. (1968). “Determinants of modes of resolving inconsistency dilemmas: a functional analysis,” in *Theories of Cognitive Consistency: A Sourcebook*, eds Abelson R. P. Aronson E. McGuire W. T. Newcomb T. M. Rosenberg M. J. Tannenbaum P. H. (Chicago, IL: Rand−McNally), 670–683.

[B44] Kenworthy J. B. Miller N. Collins B. E. Read S. J. Earleywine M. (2011). A trans-paradigm theoretical synthesis of cognitive dissonance theory: illuminating the nature of discomfort. *Eur. Rev. Soc. Psychol.* 22 36–113. 10.1080/10463283.2011.580155

[B45] Koole S. L. Rothermund K. (2011). “I feel better but I don’t know why”: The psychology of implicit emotion regulation. *Cogn. Emot.* 25 389–399. 10.1080/02699931.2010.550505 21432681

[B46] Koole S. L. Webb T. L. Sheeran P. L. (2015). Implicit emotion regulation: feeling better without knowing why. *Curr. Opin. Psychol.* 3 6–10. 10.1016/j.copsyc.2014.12.027

[B47] LaBar K. S. (2016). “Fear and anxiety,” in *Handbook of Emotions*, 4th Edn, eds Barrett L. F. Lewis M. Haviland-Jones J. M. (New York, NY: Guildford Press), 751–773.

[B48] Leippe M. R. Eisenstadt D. (1999). “A self−accountability model of dissonance reduction: multiple modes on a continuum of elaboration,” in *Cognitive Dissonance: Progress on a Pivotal Theory in Social Psychology*, eds Harmon-Jones E. Mills J. (Washington, DC: American Psychological Association), 201–232. 10.1037/10318-009

[B49] Lewis M. (2016). “Self-conscious emotions: Embarrassment, pride, shame, guilt, and hubris,” in *Handbook of Emotions*, 4th Edn, eds Barrett L. F. Lewis M. Haviland-Jones J. M. (New York, NY: Guildford Press), 792–814.

[B50] McGrath A. (2017). Dealing with dissonance: a review of cognitive dissonance reduction. *Soc. Personal. Psychol. Compass* 11:e12362 10.1111/spc3.12362

[B51] McGregor I. Newby-Clark I. R. Zanna M. P. (1999). “Remembering” dissonance: simultaneous accessibility of inconsistent cognitive elements moderates epistemic discomfort,” in *Cognitive Dissonance: Progress on a Pivotal Theory in Social Psychology*, eds Harmon-Jones E. Mills J. (Washington, DC: American Psychological Association), 325–353. 10.1037/10318-013

[B52] McGregor I. Newby-Clark I. R. Zanna M. P. (2019). “Dissonance now: how accessible discrepancies moderate distress and diverse defenses,” in *Cognitive Dissonance: Reexamining a Pivotal Theory in Psychology*, ed. Harmon-Jones E. (Washington, DC: American Psychological Association), 117–138. 10.1037/0000135-006

[B53] Mills J. (1999). “Improving the 1957 version of dissonance theory,” in *Cognitive Dissonance: Progress on a Pivotal Theory in Social Psychology*, eds Harmon-Jones E. Mills J. (Washington, DC: American Psychological Association), 25–42. 10.1037/10318-002

[B54] Mischel W. Shoda Y. (1995). A cognitive-affective system theory of personality: reconceptualizing situations, dispositions, dynamics, and invariance in personality structure. *Psychol. Rev.* 102 246–268. 10.1037/0033-295x.102.2.246 7740090

[B55] Moors A. Ellsworth P. C. Scherer K. R. Frijda N. H. (2013). Appraisal theories of emotion: state of the art and future development. *Emo. Rev.* 5 119–124. 10.1177/1754073912468165

[B56] Nolen-Hoeksema S. Wisco B. E. Lyubomirsky S. (2008). Rethinking rumination. *Perspect. Psychol. Sci.* 3 400–424. 10.1111/j.1745-6924.2008.00088.x 26158958

[B57] Nowak A. Vallacher R. R. Zochowski M. (2005). The emergence of personality: dynamic foundations of individual variation. *Develop. Rev.* 25 351–385. 10.1016/j.dr.2005.10.004

[B58] Rauthmann J. F. Gallardo-Pujol D. Guillaume E. M. Todd E. Nave C. S. Sherman R. A. (2014). The situational eight DIAMONDS: a taxonomy of major dimensions of situation characteristics. *J. Pers. Soc. Psychol.* 107 677–718. 10.1037/a0037250 25133715

[B59] Read S. J. Simon D. (2012). “Parallel constraint satisfaction as a mechanism for cognitive consistency,” in *Cognitive Consistency: A Fundamental Principle in Social Cognition*, eds Gawronski B. Strack F. (New York, NY: Guilford Press), 66–86.

[B60] Rothbart M. K. Sheese B. E. Posner M. I. (2014). “Temperament and emotion regulation,” in *Handbook of Emotion Regulation*, ed. Gross J. J. (New York, NY: Guilford Press), 305–320.

[B61] Rueda M. R. (2012). “Effortful control,” in *Handbook of Temperament*, eds Zentner M. Shiner R. L. (New York, NY: Guilford Press), 145–167.

[B62] Saarni C. (1999). *The Development of Emotional Competence.* New York, NY: Guilford Press.

[B63] Salovey P. Mayer J. D. Goldman S. L. Turvey C. Palfai T. P. (1995). “Emotional attention, clarity, and repair: exploring emotional intelligence using the trait meta-mood scale,” in *Emotion, Disclosure, and Health*, ed. Pennebaker J. W. (Washington, DC: American Psychological Association), 125–154. 10.1037/10182-006

[B64] Scherer K. R. (2009). The dynamic architecture of emotion: evidence for the component process model. *Cogn. Emot.* 23 1307–1351. 10.1080/02699930902928969

[B65] SFS (2003). *The Act Concerning the Ethical Review of Research Involving Humans (SFS 2003:4).* Stockholm: The Swedish Ministry of Education and Cultural Affairs.

[B66] Sheppes G. (2014). “Emotion regulation choice: theory and findings,” in *Handbook of Emotion Regulation*, ed. Gross J. J. (New York, NY: Guilford Press), 126–139.

[B67] Sheppes G. Scheibe S. Suri G. Gross J. J. (2011). Emotion-regulation choice. *Psychol. Sci.* 22 1391–1396. 10.1177/0956797611418350 21960251

[B68] Sherman S. J. Gorkin L. (1980). Attitude bolstering when behavior is inconsistent with central attitudes. *J. Exp. Soc. Psychol.* 16 388–403. 10.1016/0022-1031(80)90030-x

[B69] Simon L. Greenberg J. Brehm J. (1995). Trivialization: the forgotten mode of dissonance reduction. *J. Pers. Soc. Psychol.* 68 247–260. 10.1037/0022-3514.68.2.247

[B70] Skinner E. A. Edge K. Altman J. Sherwood H. (2003). Searching for the structure of coping: a review and critique of category systems for classifying ways of coping. *Psychol. Bull.* 129 216–269. 10.1037/0033-2909.129.2.216 12696840

[B71] Steele C. M. Liu T. J. (1983). Dissonance processes as self-affirmation. *J. Pers. Soc. Psychol.* 45 5–19. 10.1037/0022-3514.45.1.5

[B72] Stone J. Aronson E. Crain A. L. Winslow M. P. Fried C. B. (1994). Inducing hypocrisy as a means of encouraging young adults to use condoms. *Pers. Soc. Psychol. Bull.* 20 116–128. 10.1177/0146167294201012

[B73] Tamir M. John O. P. Srivastava S. Gross J. J. (2007). Implicit theories of emotion: affective and social outcomes across a major life transition. *J. Pers. Soc. Psychol.* 92 731–744. 10.1037/0022-3514.92.4.731 17469955

[B74] Vaidis D. C. Bran A. (2018). Some prior considerations about dissonance to understand its reduction: comment on McGrath (2017). *Soc. Pers. Psychol. Compass* 92 1–13. 10.1111/spc3.12411

[B75] Vallacher R. R. Van Geert P. Nowak A. (2015). The intrinsic dynamics of psychological process. *Curr. Direct. Psychol. Sci.* 24 58–64. 10.1177/0963721414551571

[B76] Watkins E. (2004). Appraisals and strategies associated with rumination and worry. *Pers. Ind. Diff.* 37 679–694. 10.1016/j.paid.2003.10.002

[B77] Webb T. L. Miles E. Sheeran P. (2012). Dealing with feeling: a meta-analysis of the effectiveness of strategies derived from the process model of emotion regulation. *Psychol. Bull.* 138 775–808. 10.1037/a0027600 22582737

[B78] Weick K. E. (1968). “The panglossian world of self-justification,” in *Theories of Cognitive Consistency: A Sourcebook*, eds Abelson R. P. Aronson E. McGuire W. T. Newcomb T. M. Rosenberg M. J. Tannenbaum P. H. (Chicago, IL: Rand McNally), 706–715.

[B79] Wicklund R. A. Brehm J. W. (1976). *Perspectives on Cognitive Dissonance.* Hillsdale, NJ: Erlbaum.

[B80] Zanna M. P. Aziza C. (1976). On the interaction of repression-sensitization and attention in resolving cognitive dissonance. *J. Pers.* 44 577–593. 10.1111/j.1467-6494.1976.tb00139.x 1011068

